# The Impact of the Wavelet Propagation Distribution on SEIRS Modeling with Delay

**DOI:** 10.1371/journal.pone.0098288

**Published:** 2014-06-09

**Authors:** Ofosuhene O. Apenteng, Noor Azina Ismail

**Affiliations:** Department of Applied Statistics, Faculty of Economics & Administration, University of Malaya, Kuala Lumpur, Malaysia; Centers for Disease Control and Prevention, United States of America

## Abstract

Previous models of disease spread involving delay have used basic SIR (susceptible – infectious – recovery) formulae and approaches. This paper demonstrates how time-varying SEIRS (S – exposed – I – R – S) models can be extended with delay to produce wave propagations that simulate periodic wave fronts of disease spread in the context of population movements. The model also takes into account the natural mortality associated with the disease spread. Understanding the delay of an infectious disease is critical when attempting to predict where and how fast the disease will propagate. We use cellular automata to model the delay and its effect on the spread of infectious diseases where population movement occurs. We illustrate an approach using wavelet transform analysis to understand the impact of the delay on the spread of infectious diseases. The results indicate that including delay provides novel ways to understand the effects of migration and population movement on disease spread.

## Introduction

There is very little understanding of how disease spread is affected by population migration. When modeling the propagation of disease, it is important to model not only individual infections but also the spread of infection from populations of infected individuals to other populations [1]. In particular, if the disease is fatal, what matters is the likelihood of the disease spreading to new individuals before the currently infected individuals die. Mathematical models have been developed for use in the dynamic modeling of disease spread [2], [3], such as the SIR model, which represents the state changes among the members of the susceptible (*S*), infected (*I*) and recovered (*R*) populations. The goal of such models is to understand the disease spread patterns and to predict the outcomes of introducing public health intervention measures to minimize the spread of disease. Such models must take into account the difference in time between becoming exposed (*E*) to infection and becoming infected (hence, SEIR) as well as between becoming infected and dying (‘delays’). A wide variety of such approaches of modeling disease with delay have been formulated. Some of these delay concepts are strongly parametric (specific distributions), while others are nonparametric (general distributions). However, there is very little understanding of how the SEIR models are affected by delay [4].

A delay in many population models considers that the transmission dynamic behavior of the disease at time *t* can destabilize the equilibrium [5]. There are two types of delay: a discrete or fixed delay and continuous or distributed delay [6]. In models with a discrete or fixed delay, the dynamic behavior of the disease depends on the state at time 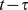, where 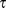 is a fixed constant [7]. For example, the number of newborns at time 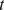 depends on the state of population at time 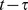, where 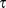 the period of pregnancy. In the case of a continuous or distributed delay, the dynamic behavior of the model at time 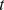 depends on the states during the entire period prior to time 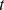. Both types of delays produce outputs at the end of each time step that can be interpreted as a signal, or function, of the model. This signal or function can be analyzed further to extract the features, such as frequency and amplitude, which can be mapped onto interpretable properties of the model. Starting a model with two different sets of parameter values can lead to two different signals over time, which, upon further analysis, can be mapped back to the effects that the parameter values have on the model. Such signals can include oscillations and waves if periodicity (a function that repeats its values at regular intervals) emerges from the model. The periodicity can itself be analyzed using wavelet or Fourier transforms.

The advantage of the wavelet transform of a signal over the Fourier transform is the ability of wavelet transforms to capture local information (i.e., feature changes in space or time), whereas Fourier transforms capture global features (e.g., harmonic components of the entire signal). That is, wavelet transforms are localized in both time and space, and can be useful for identifying parts of the signal that are local in terms of the geographical location. Wavelet transforms have many uses in the areas of wave propagation, data compression, signal processing, image processing and pattern recognition [8], [9]. In this paper, we demonstrate how cellular automata supplemented with wavelet transforms can be used to implement a SEIRS model that attempts to simulate the spread of disease locally in both time and space, where space refers to the delay from exposed individuals to infectious individuals.

### Description of the Model

In this section, we introduce cellular automata (CA) for modeling geographical distributions. CA has a significant role in epidemic modeling because each individual or cell, or a small region of space, updates itself independently, allowing for the concurrent development of several epidemic spatial clusters. The new state of a cell is based on its current state and the current states of its surrounding cells as well as on some shared rules of change.

The SEIRS model used here is specified as follows. Let 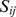 be the number of susceptible individuals 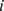 in the population at some patch 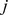 at time 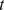. Let 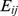 represent the number of susceptible individuals who become exposed (infected but not infectious) [10] at that patch. An individual remains in the exposed class or the exposed state for a certain latent period before becoming infected [11]. Let 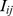 denote the number infected at that patch and 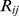 denote the number recovered at that patch. If 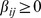 is the effective contact rate per individual per unit of time at a patch, we can use the Law of Mass Action assumption that the rate of change from being susceptible to being exposed and finally to natural death is proportional to the population number in each state, with a rate constant 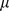. The assumption is that 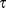 is the delay time (days), which is a constant. Adopting the approach of Yan and Liu [12], the probability that an individual survives the latent period to become infected at 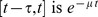. Additionally, the number of susceptible individuals who becomes exposed at time 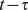 is 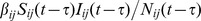. The difference between 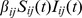 and 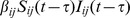 is that former is the exposed state and the latter is the infected state, which leads to a deterministic model of 
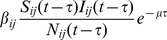
.

The task for the wavelet transform is to identify, from the composite signal formed from the outputs of the model at each time step (to be described in more detail below), any local temporal and spatial periodicities that emerge.

### Rules for Disease Spread

The rules described below [13] determine the state transitions of individual cells in the CA for the SEIRS model at each time step and will incorporate other probabilistic parameters.

**Figure 1:**
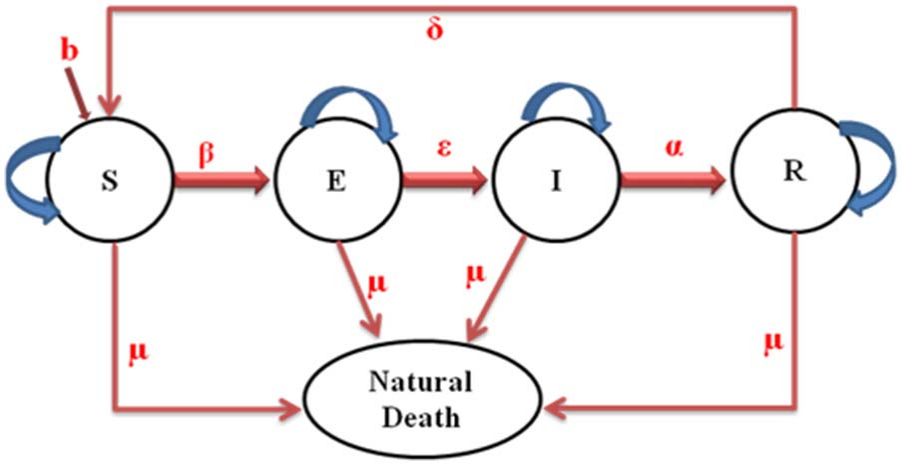
Movement with all exposed class in SEIRS Model. Depicts the flow chart of the SEIRS model.

- A cell changes its state from *susceptible* to *exposed* 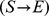 when it comes in contact with an infected cell within its defined neighborhood.
- A cell changes its state from *exposed* to *infectious 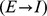
* after being in the state 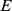 for a given 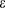, which is the transition time.
- The state of the cell changes from *infectious* to *recovery* 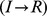 after being in the state 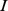 for a given time 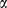. In our model, we assume that for every cell, the amount of time 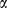 elapsed from the *infectious* cell state to the *recovery* cell state. In state 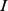, the cells are capable of passing on the infection to neighboring cells.
- The cell remains in state 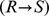, signifying complete recovery for some time 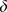, which is the transition time between the recovery state and the susceptible state, as shown in Fig. 1.

These CA rules can be described by a concrete expression using the classic SEIRS models based on differential equations. The set of ordinary differential equations corresponding to the CA model is described in the following.
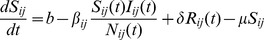
(1)

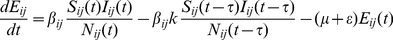
(2)

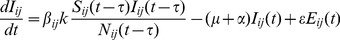
(3)

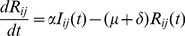
(4)where 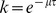




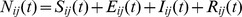



It is assumed that the population sizes of each patch are identical and remain constant. This corresponds to assuming that the population birth and death rates, denoted by 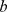 and 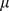 respectively. Individuals are born susceptible and can acquire infection from infective individuals, at which point they enter the exposed state. After a latent period, lasting an average of 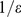 time units, individuals are infectious for an average of 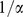 time units. Since the population is subject to a disease-independent mortality rate, 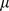, the mortality-corrected average duration of infectiousness is 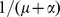, and the exposed individuals who will become infectives is 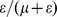. This formulation of the model assumes that there is migration rate, 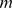 into exposed class to ascertain the period at which all exposed class migrate to infected class.

As mentioned above, the infection is modeled as the contact between an infected individual and susceptible individuals in the neighborhood, either as a deterministic or as a probabilistic process, depending on how the conditions on the arcs of the model in Figure 1 are realized. For our experiments below, we use probabilistic conditions.

### Simulation Scenario

In this paper, 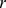 is the radius used for the neighborhood around the cardinality point within which the nearest cell is infected. The 1^st^ order Moore neighborhood was defined as the 8 nearest neighbors, i.e., 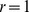. The degree of infectiousness was used to assess the probabilistic distribution that the current cell is infected in the next time step. The disease will propagate through the landscape based on a set of probabilities of state transitions [14]. Before an infected cell disperses (migrates), it updates independently, based on the variable location of the neighboring cells, with probability 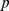.
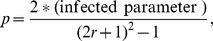
(5)where 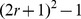 is the cardinality of the neighboring cell.

Each infected individual can enter the cell from the cardinality of the neighboring cell with the same probability 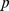, whereas there are a number of new infected that arrive in the cell from the neighboring cells. Finally, the state of the cell after a complete transition at time 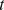 is updated by the iteration number (time), that is, 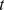 is increased by 1 to become time 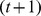.

### Stability Analysis

An important equilibrium point for any disease model is the disease-free equilibrium (DFE). The stability of the DFE is especially important because it determines whether a disease is capable of attacking an entire population beyond the prior expectation (epidemic or pandemic) [15]. The reproduction number 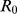 is a threshold value or number that determines the stability of the DFE [16]. The reproduction number is the expected number of secondary cases produced by a typical infection in a completely susceptible population [17]. If 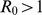, an epidemic occurs; if 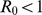, an epidemic does not occur; and if 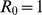, a change of stability occurs. 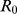 can be calculated as 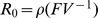, the spectral radius 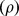 of the next generation matrix 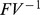. The 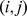 is the number of entries of matrix 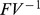, which represents the number of new infections in compartment 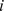 due to an infected individual being introduced into compartment 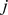 [17], [18].

Let 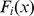 be the rate at which newly infected individuals (transmitted rate) enter compartment 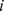
_,_ and let 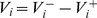 denote the transfer of individuals out of 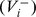 and into 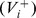 the 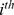 compartment. With this interpretation, we write a matrix 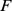 that defines the rate of new infections in different compartments, differentiated with respect to 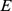 and 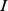, and then evaluated at the disease-free equilibrium. An equilibrium solution with 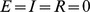 and 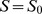, where 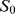 is any positive solution of 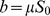. Therefore, this will be locally stable and hence a disease free equilibrium, 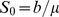. We assume that, this to be the case, evaluating the derivatives of 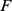 and 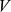 at 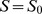, 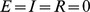, we get the following expressions for 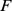, 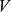 and the spectral radius respectively.
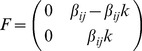
(6)


Now, we also write 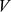 that defines the rate of transfer of infected from one compartment to another 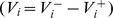.
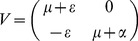
(7)

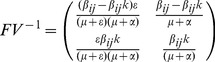
(8)The reproduction number *R_0_* is given by the dominant eigenvalue (or spectral radius) of 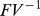.

Then,
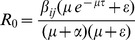
(9)



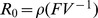
, where 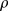 denotes the spectral radius.

If 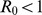, then the DFE is globally asymptotically stable; if 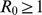, then the DFE is unstable [16].

### Why Use Wavelet Analysis?

The wavelet transform is a function that is an improved technique of implementing the Fourier transformation. In the time-frequency analysis of a set of data, the classical Fourier transform analysis is inadequate due to a lack of any local information contained in the data. The use of wavelet analysis describes the pattern, trends, and the structures that might be overlooked in the raw data. The usefulness of wavelets in data analysis is very clear, particularly in the field of statistics, where large and cumbersome data sets are prevalent. Wavelet analysis is used here to accomplish the following tasks:

- to analyze the frequency of the spread of the disease; and
- to understand the impact of the delay in the spread of an infectious disease.

These two tasks will be accomplished by analyzing the frequency and interpreting the exposed as a function of the delay.

Let 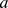 be certain class of functions and 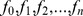 be the frequencies of a simple function such that each 
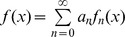
 for some coefficients 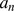. We will consider a function in the Laplace transform 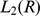, i.e.,
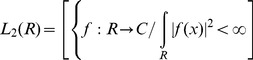
(10)In that case, we consider a wavelet function 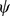, such that
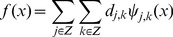
(11)where 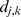 are the wavelet coefficients and 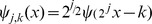 are the translated and scaled version of the wavelet 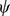.

Let 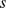 represent each compartment and 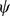 be the wavelet. Then, the wavelet coefficient of 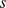 at scale 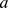 and position 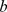 is defined by:
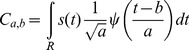
(12)Because 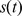 is discrete, we will use a piecewise constant interpolation of the 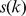 values, where 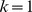 to *length*(*s*).

### Simulation Setup

Cellular automata are found to have received extensive academic study as one of the mathematical tools for successfully modeling the spread of diseases. Because there are a number of cellular automata used in the Matlab programming environment [19], Matlab was chosen as the implementation tool here.

## Experiment and Results

We constructed cellular automata in Matlab to implement the model described by equations (1)–(4) stated in the previous section. The experiments were performed on a 100×100 cell spaces. We assumed that twenty infected individuals were introduced into the population of 100×100 cells, which corresponds to ten-thousand individuals. Table 1 summarizes the parameter values used. Blue, light green, red and light blue squares correspond to the values of 0, 0.5, 1 and 0.1, respectively, with the values of 0, 0.5, 1 and 0.1, denoting susceptible, exposed, and infected and recovery, respectively. A total of 365 iterations were simulated, with one iteration representing one day. The variables and parameters are shown in Table 1.

**Table 1:** Simulation Protocol.

Events	Model Prediction	CA model
Environment	Spatial (landscape)	Spatial (landscape)
Susceptible	9980 people	9980 people
Exposed	Nil	From model prediction
Infected	20 people	20 people
Recovery	Nil	From model prediction
Natural death rate	0.0005 yr^−1^	0.0005 yr^−1^
Incubation period, *_τ_*	7 days	7 days
No. of simulation	365 iterations	365 iterations
Infected Factor, *β*	0.025	0.25

**Figure 2:**
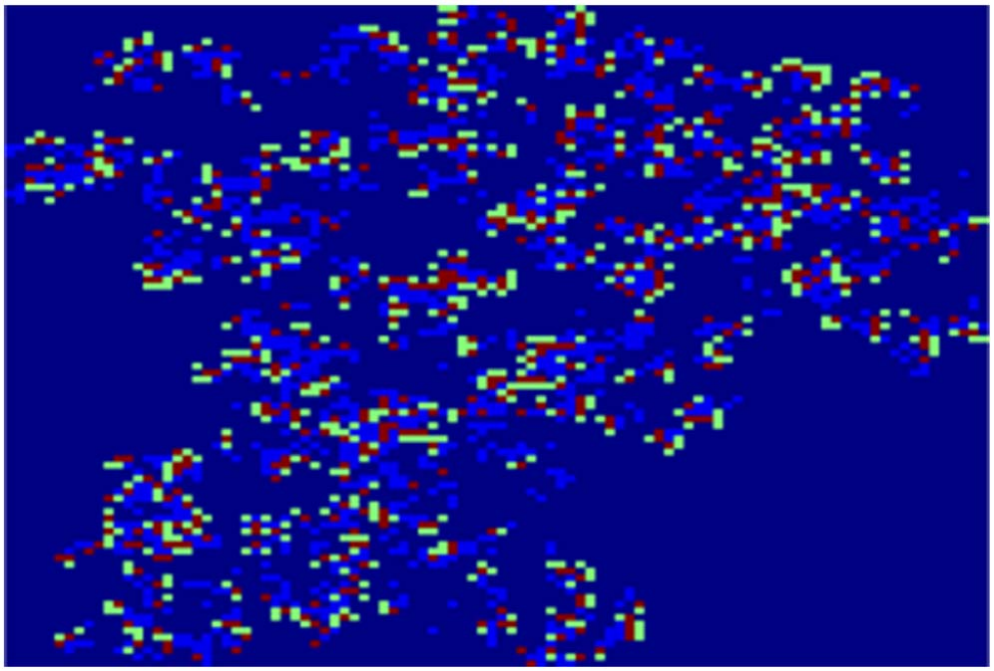
A dynamic spread process. Depict the geographical distribution of the propagation of disease spread clusters for 40.

**Figure 3:**
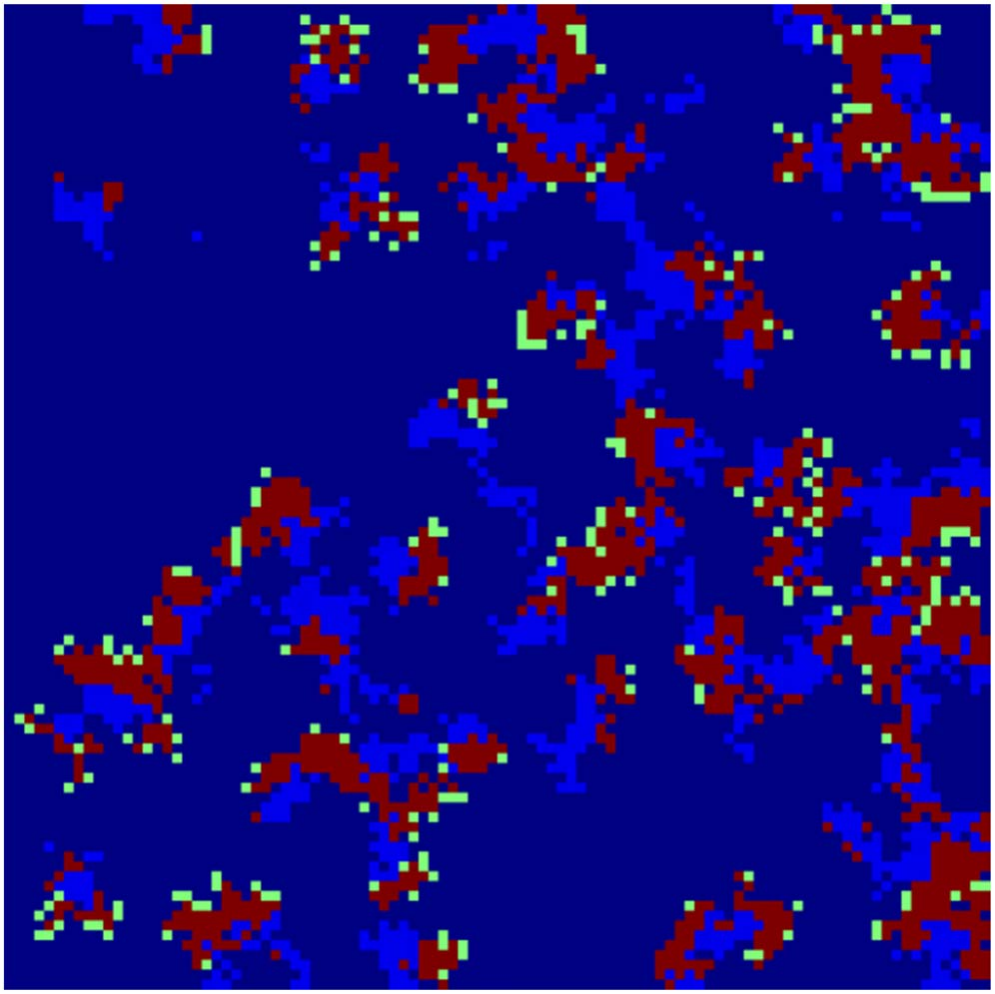
A dynamic spread process. Depict the geographical distribution of the propagation of disease spread clusters for 65 simulations.

Figure 2 and 3 depict the geographical distribution of the propagation of disease spread clusters for 40 and 65 simulations, respectively.

The colors dark blue, green, red and light blue represent healthy individuals (susceptible), infected individuals (exposed), infectious individuals and recovery individuals, respectively. We can see the breakdown of an initially homogenous spread-of-disease pattern. As the phase separation progresses, a persistent compact spread of disease is formed, as shown in Figure 2. Figure 3 shows the further progression of the phase separation, in which a persistent compact spread of disease is formed (infectious), surrounded by exposed individual populations.

**Figure 4:**
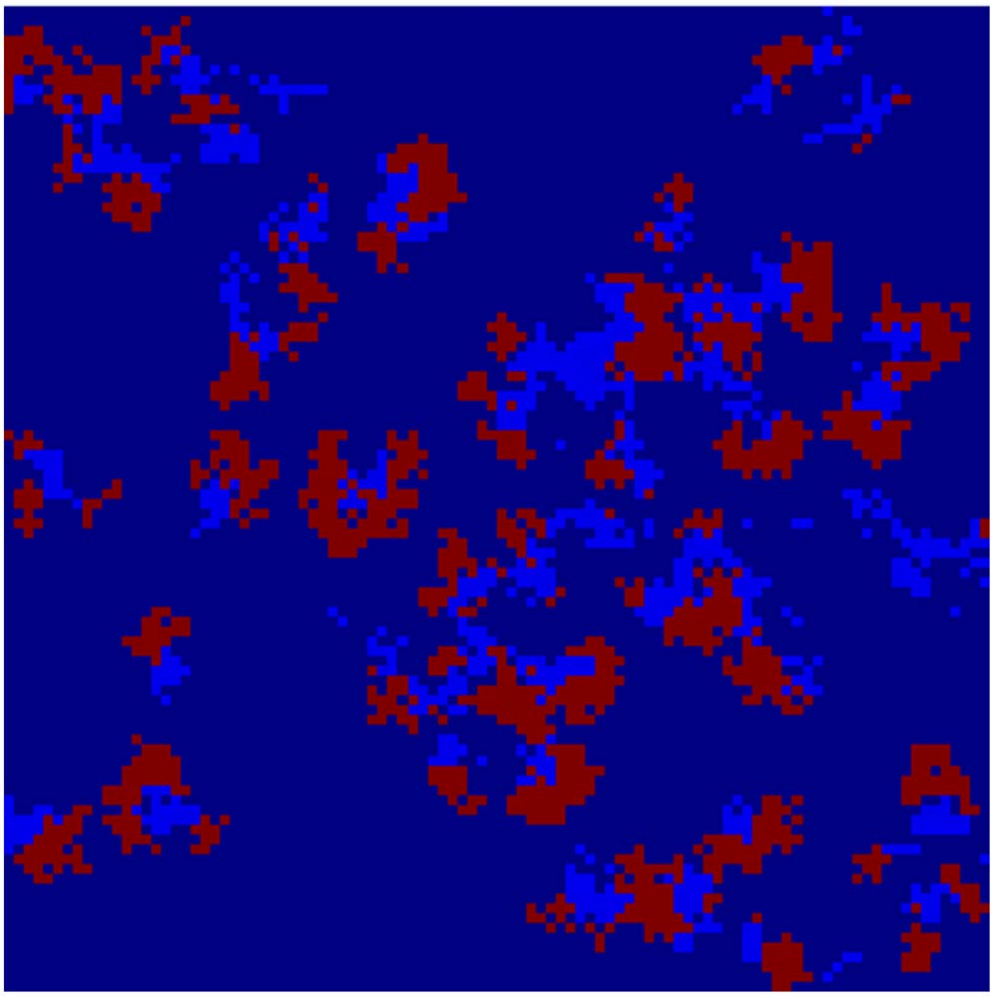
A dynamic spread process without exposed state. Depicts how the propagation of the disease spread clusters proceeds in geographical distribution using 365 simulations. The model indicates that there is increasing volatility in the susceptible population after 240 days due to outgoing moves from the susceptible to the exposed population (Figure 5A).

**Figure 5:**
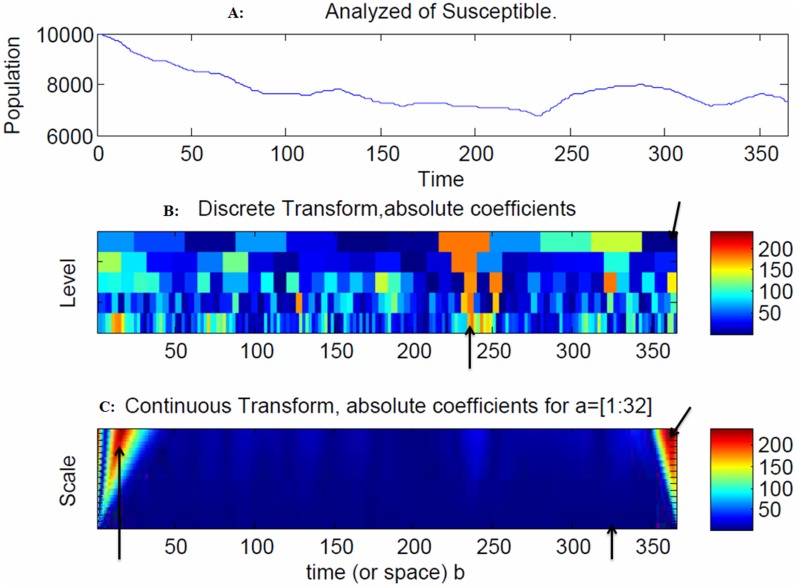
Snapshot of the susceptible population. The model indicates that there is increasing volatility in the susceptible population after 240 days due to outgoing moves from the susceptible to the exposed population (Figure 5A). The spread of the disease in the discrete transform exhibited peaks at 230 days and 360 days (Figure 5B), as indicated by the arrows. Slower and faster frequencies were found at the initial stages as well as the final stages at 10 days and 360 days, as indicated by the arrows. The blue regions denote the probability densities. For example, at 330 days, there was an indication of a slower frequency that resulted in a faster spread of the disease (Figure 5C) due to the large coefficients in the continuous wavelet transform.

Figure 4 depicts how the propagation of the disease spread clusters proceeds in geographical distribution using 365 simulations. The model indicates that there is increasing volatility in the susceptible population after 240 days due to outgoing moves from the susceptible to the exposed population (Figure 5A).

The colors of dark blue, red and light blue represent healthy individuals (susceptible), infectious individuals and recovery individuals, respectively. As the phase separation proceeds, a persistent compact spread of disease is formed (infectious and recovery) within the population. The spread of the disease in the discrete transform exhibited peaks at 230 days and 360 days (Figure 5B), as indicated by the arrows. Slower and faster frequencies were found at the initial stages as well as the final stages at 10 days and 360 days, as indicated by the arrows. The blue regions denote the probability densities. For example, at 330 days, there was an indication of a slower frequency that resulted in a faster spread of the disease (Figure 5C) due to the large coefficients in the continuous wavelet transform.

**Figure 6:**
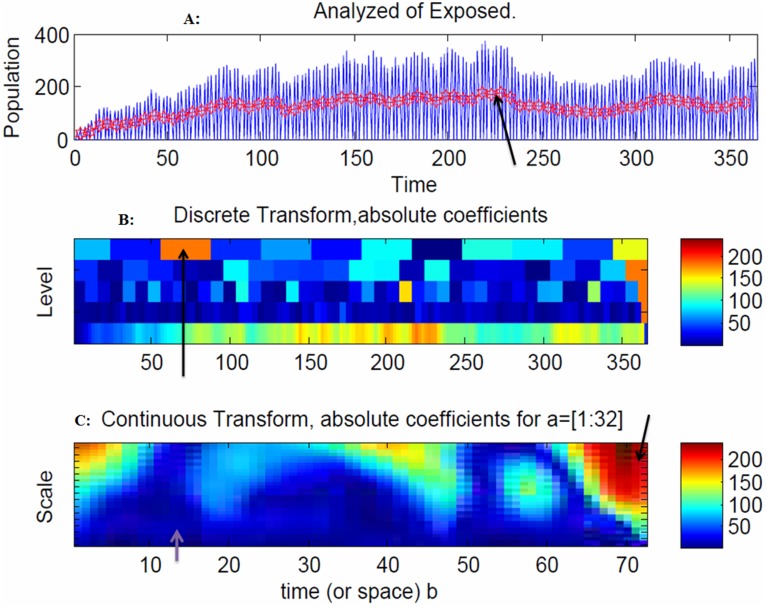
Snapshot of the exposed population. The model indicates that there is an increased volatility in the exposed population after 200 days, due to the outgoing moves from the exposed population to the infectious population (Figure 6A), and an increased volatility in the infectious population after 240 days, due to the outgoing moves from the infectious population to the recovery population (Figure 7A). The spread of the disease in the discrete transform reached a peak at 70 days due to the large coefficients (Figure 6B). In the continuous wavelet transform at 72 days, there were no exposed individuals (Figure 6C) because they moved to the infectious stage.

**Figure 7:**
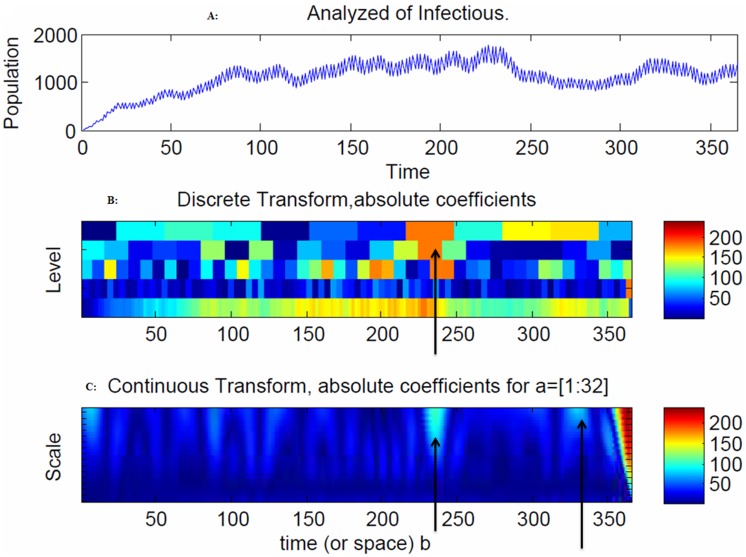
Snapshot of the exposed population. The spread of the disease in the discrete transform peaked at 240 days (Figure 7B), and at 240 days and 280 days (Figure 7C) due to large coefficients. The range of frequencies used in averaging is indicated by the arrow at 240 days, which corresponds to the peak of the disease spread (Figure 7C).

The model indicates that there is an increased volatility in the exposed population after 200 days, due to the outgoing moves from the exposed population to the infectious population (Figure 6A), and an increased volatility in the infectious population after 240 days, due to the outgoing moves from the infectious population to the recovery population (Figure 7A).

The spread of the disease in the discrete transform reached a peak at 70 days due to the large coefficients (Figure 6B). In the continuous wavelet transform at 72 days, there were no exposed individuals (Figure 6C) because they moved to the infectious stage. The spread of the disease in the discrete transform peaked at 240 days (Figure 7B), and at 240 days and 280 days (Figure 7C) due to large coefficients. The range of frequencies used in averaging is indicated by the arrow at 240 days, which corresponds to the peak of the disease spread (Figure 7C).

**Figure 8:**
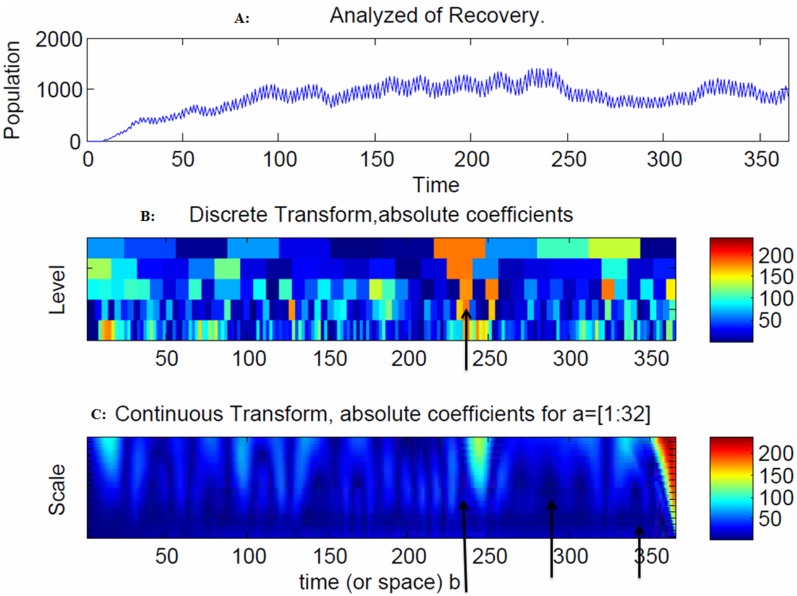
Snapshot of the infected population. The model indicates that there is increasing volatility in the recovery population after 240 days due to the outgoing moves from the recovery population to the natural death population (Figure 8A). The spread of the disease in the discrete transform peaked at 240 days (Figure 8B) and at 230, 250 and 340 days due to large coefficients (Figure 8C). The range of frequencies used in averaging is indicated by the arrow at 250 and 362 days, which correspond to the peaks of the disease spread (Figure 8C).

**Figure 9:**
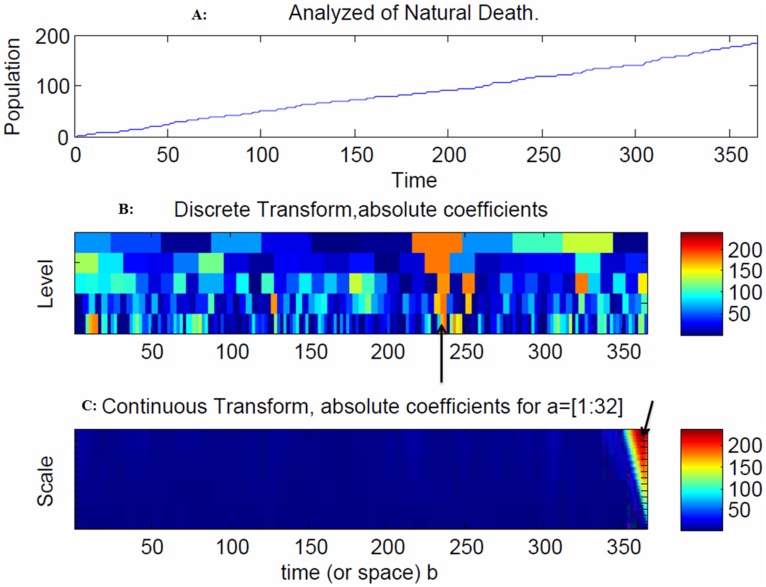
Snapshot of the recovery population. An increase in natural deaths was observed after 50 days due to the outgoing moves from the infectious and recovery populations to the natural death population (Figure 9A). The range of frequencies used in averaging is indicated by the arrow at 362 days, which corresponds to the peak of the disease spread (Figure 9C).

The model indicates that there is increasing volatility in recovery after 240 days due to the outgoing moves from the infectious population to the recovery population (Figure 8A). In addition, an increase in natural deaths was observed after 50 days due to the outgoing moves from the infectious and recovery populations to the natural death population (Figure 9A).

The model indicates that there is increasing volatility in the recovery population after 240 days due to the outgoing moves from the recovery population to the natural death population (Figure 8A). The spread of the disease in the discrete transform peaked at 240 days (Figure 8B) and at 230, 250 and 340 days due to large coefficients (Figure 8C). The range of frequencies used in averaging is indicated by the arrow at 250 and 362 days, which correspond to the peaks of the disease spread (Figure 8C). The spread of the disease in the discrete transform were at a peak at 240 days (Figure 9B). The range of frequencies used in averaging is indicated by the arrow at 362 days, which corresponds to the peak of the disease spread (Figure 9C).

**Figure 10:**
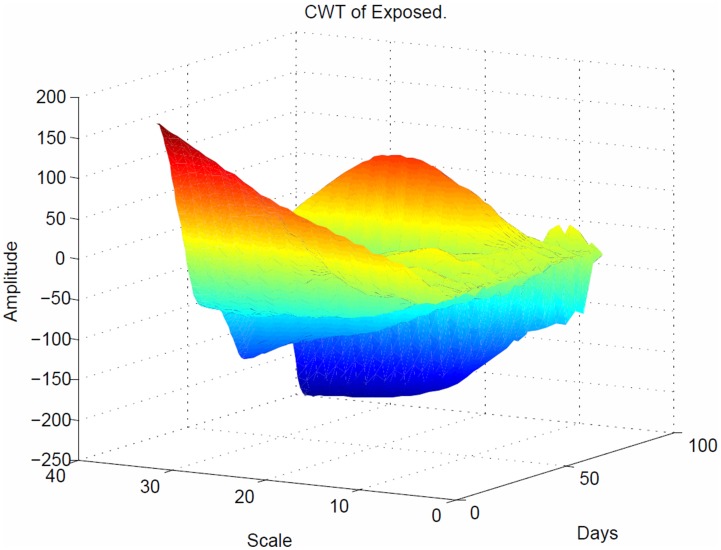
Sinusoidal response of the exposed population in 3 dimensions. The CWT coefficients are large at scales near the frequencies of the exposed waves and clearly depict the sinusoidal pattern in the CWT coefficients at these scales for the exposed population.

**Figure 11:**
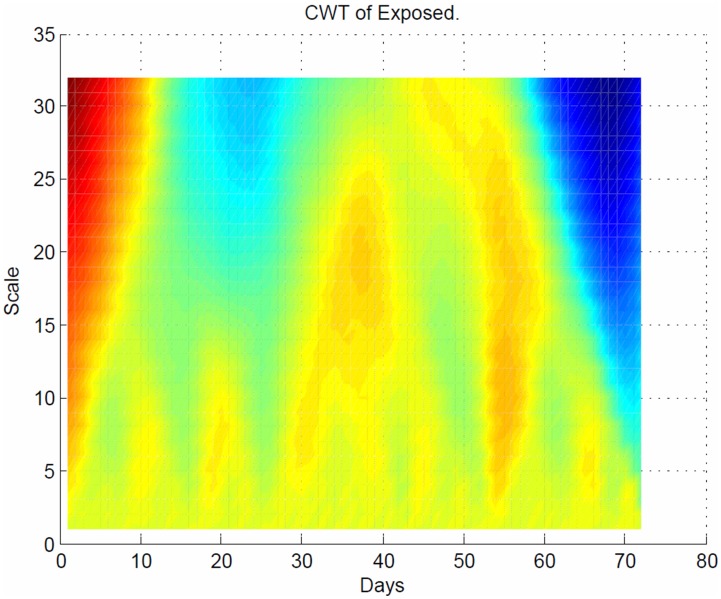
Sinusoidal response of the exposed population. Plots the same transform from Figure 10 but with different angle for better visualization, depicts the time period at which all the individuals who were exposed to disease moved to infectious population.

As shown in Figure 10, the CWT coefficients are large at scales near the frequencies of the exposed waves and clearly depict the sinusoidal pattern in the CWT coefficients at these scales for the exposed population. Figure 11 plots the same transform from a different angle for better visualization.

The sinusoidal wave amplitude is the height of the crest, and the frequency is the number of oscillations per second. Hence, the amplitude remains the same for any change in frequency. The maximum and minimum are both at 225 Hz, which demonstrates that the spread of the disease in the exposed population was at the peak state (Figure 10). In this plot, the value of each (x, y) coordinate represents the strength of the spread of the disease between the coordinates. The strong interaction between the 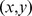 coordinates is 0.026 Hz (Figure 11). As seen in Fig. 6C, the arrow at 70 days indicated the faster frequency, which resulted in a slower spread of the disease.

## Conclusion

We have developed a mathematical disease propagation model of a susceptible-infected-recovery (SIR) type extended to a four compartmental epidemiological model with a susceptible-exposed-infectious-recovery-susceptible (SEIRS) with exposed class moved as the spread of an infectious disease in a population with a delay. The discrete and continuous transforms demonstrate where and how the propagation of disease was dominant in the population. The interactions between the infected and the susceptible lead to an exposed stage of the disease, which agrees with global dynamics behaviors for a new delay SEIR epidemic disease model proposed by Meng and Chen et al. [4]. Wavelets are very important for detecting abrupt changes in the distribution of disease spread [20]. These abrupt changes occur from one compartment to another (susceptible, exposed, infectious, recovery, and susceptible). These changes produced relatively large wavelet coefficients centered on the discontinuity at all scales. The location of the discontinuity based on the CWT coefficients is obtained at the smallest scales. This selection for shorter infection period was also reported in [13]. Note that, due to the stochastic nature of the model parameters, the introduction of the dead period is only a transient phenomenon. As shown in Figure 7, the number of individuals who were infectious to the disease was much higher at the beginning of the spread of the disease. An infection will only cause a new infection if the individual is still exposed at the time of infection. Alimadad and Dabbaghian et al. [21] also reported that an individual becomes exposed to an infection when a randomly chosen neighbor individual is susceptible to it. The dominant order of the frequencies indicates that a delay in the transition between exposed to infectious states occurs after the peak points in the different time intervals. The detection of these discontinuities (delays) was associated with the speed of the spread of the disease and is detectable in the frequency and phase of the CWT. The disease stopped spreading in the exposed state at 72 days (Figure 11) but continued to spread in the susceptible, infectious and recovery populations after 240 days. The spread of the recovery population continued to persist, as shown in Figure 4, 5, 7, 8 and 9. This behavior implies that: a) the higher the frequency, the lower the spread of the disease, and b) the faster the spread of the disease, the slower the frequency. The discrete and continuous transforms demonstrate where and how the propagation of disease was dominant in the population. Therefore, we have demonstrated how to model the delay through exposure and the effects of the delay on a movement of exposed state to infectious population. Finally, the proposed model will be tested against real world data to test its fitness and to evaluate its accuracy as well as to determine the utility of the cellular automata model.
